# Adolescent suicide trends in Brazil (2000–2022): An ecological analysis by sex, age, and suicide methods

**DOI:** 10.1371/journal.pone.0309505

**Published:** 2025-07-18

**Authors:** Rafael Bello Corassa, Rayone Moreira Costa Veloso Souto, Maria Carmen Viana, Otaliba Libânio Morais Neto

**Affiliations:** 1 Instituto de Patologia Tropical e Saúde Pública, Universidade Federal de Goiás (UFG), Setor Universitário, Goiânia/GO, Brasil; 2 Departamento de Medicina Social, Universidade Federal do Espírito Santo (UFES), Bonfim, Vitória/ES, Brasil; Oswaldo Cruz Foundation (FIOCRUZ), BRAZIL

## Abstract

**Introduction:**

Suicide is the 3^rd^ leading cause of death among Brazilian adolescents. Despite the rising rates, national studies on suicide trends still face methodological limitations and data quality issues. This study aimed to investigate adolescent suicide trends in Brazil.

**Methods:**

An ecological time-series study was conducted to analyse suicide (ICD-10: X60-X84, Y87.0) trends among 10–19-year-old adolescents from 2000 to 2022, stratified by sex, age group, and self-harm method. Garbage code redistribution was applied to correct misclassified underlying causes of death. Corrected suicide rates were estimated and time trend analysis was performed using joinpoint regression.

**Results:**

After the redistribution, 22,591 adolescent suicides were identified. Rates increased by 120% over the period, with a sharp rise starting in 2013 (APC: 7.7%; 95%CI: 6.2–9.3), particularly among 10–14-year-old girls after 2011 (APC: 10.6%; 95%CI: 8.2–13.0). Male rates were nearly twice as high than female rates, but 10–14-year-old female rates surpassed males after 2018. Hanging was the most frequent method (65.9%), followed by poisoning (15.7%) and firearms (10.8%). Hanging-related deaths increased across all subgroups, and the downward trend in firearm-related suicides reversed after 2010 (APC: 3.0%; 95%CI: 0.6–5.4).

**Conclusions:**

The increasing trend of adolescent suicides in Brazil, notably among young females and through more lethal methods, suggests a deterioration of adolescents’ mental health and well-being. The findings underscore the need for comprehensive suicide prevention strategies addressing individual, social, and environmental factors, and the importance of accurate data for policymaking. The study calls for urgent action to tackle the growing problem of adolescent suicides in Brazil.

## Introduction

Suicide is a serious public health problem and a significant cause of premature death worldwide [1]. Since the World Health Organization (WHO) published the report “Preventing suicide: A global imperative”, there has been an increasing concern about this issue, prompting its inclusion in the United Nations’ Sustainable Development Goals [2].

Globally, suicides account for over 700,000 annual deaths, of which more than 75% occur in low and middle-income countries [1–3]. The situation is particularly challenging among the adolescent and youth population [1]. Globally, data from the Global Burden of Disease (GBD) 2021 study ranks suicide as the 20th leading cause of death among the general population, while among teenagers, it ranks 2nd, surpassed only by road traffic injuries [3].

In Brazil, GBD 2021 data ranks suicide as the 22nd leading cause of death among the general population, and the 3rd leading cause among adolescents, surpassed only by interpersonal violence and road traffic injuries [3]. Furthermore, studies have demonstrated increasing trends in adolescent suicide rates in the country [4–6].

An analysis of adolescent suicide rates in Brazil, from 2000 to 2015, showed an increasing trend in these deaths among males, while female rates remained stable [4]. Moura et al. [5], in a national study, observed an increasing trend in adolescent suicide mortality in both sexes and in the age groups of 10 to 14 and 15 to 19 years between 2010 and 2018. In another study, Silva et al. [6] noted a rise in suicide rates among male adolescents in the Northeast region of Brazil between 2001 and 2015. Meanwhile, female teenage suicide rates in the 10 to 14 age group increased between 2000 and 2007, followed by a downward trend, while 15 to 19 age group rates remained stable throughout the period.

Despite providing relevant data and significant methodological advancements, studies on the magnitude and trends of adolescent suicides in Brazil still have important gaps and limitations. National studies have analysed teenage suicides by strata of either age or gender [4,5], but have not investigated trends in recent years or examined suicide methods. In addition, these studies have not considered limitations regarding variations in data quality through the years, which may influence overall trends. Consequently, more detailed and in-depth analyses are necessary.

Understanding the national trend of adolescent suicides is essential to estimate the true extent of the phenomenon, identify priorities, design and implement prevention policies and interventions. Therefore, this study aimed to investigate the trends in suicide mortality among adolescents in Brazil, considering stratifications of gender, age group, and suicide method.

## Methods

An ecological time-series study of adolescent (aged 10–19 years) suicide mortality in Brazil, from 2000 to 2022, was conducted. Mortality data were obtained from the Mortality Information System (SIM) of the Ministry of Health. Population data were obtained from population projections by the Brazilian Institute of Geography and Statistics (IBGE) from 2000 to 2070 [7].

Previous analyses have shown large improvements in mortality data quality in Brazil between 2000 and 2015 [8]. However, there has been a sharp increase in the proportion of deaths due to undetermined intent in the Mortality Information System (SIM) in recent years [9,10], which may limit the understanding of the phenomenon and influence the analysis of recent time trends. Therefore, in order to adjust suicide estimates for the variations in data quality, redistribution of deaths classified as garbage codes (GCs) was conducted, as recommended by previous studies [11,12].

The redistribution of GCs was conducted by adapting the procedures proposed by Soares Filho et al. [11] and compiled by Teixeira [13]. The first step consisted of mapping ICD-10 GCs to be redistributed and target groups of causes (S1 Appendix in Supplementary Table 1). Step two consisted of proportional redistribution of records with missing data on age and sex. Step three consisted of redistribution of GCs. Redistribution of GCs was conducted by combining proportional and empirical redistribution of groups of ICD-10 codes, using predefined weights calculated based on the ‘Data for Health’ (60 Cities Project) [14].

The ‘Data for Health’ initiative [14] was a national project, conducted by the Ministry of Health, that provided training and technical support to investigate deaths classified as garbage codes, in order to improve mortality data quality. The project focused on 60 cities that concentrated 35% of all deaths, distributed across all five national regions. Redistribution weights for each group of ICD-10 codes are available in S1 Appendix in Supplementary Table 2.

Based on the corrected death counts, national suicide mortality rates were estimated for the years 2000 to 2022, stratified by gender, age group, and suicide method. Based on previous research [15–18], suicides were defined as deaths with the underlying cause coded as X60-X84 or Y87.0 in the International Statistical Classification of Diseases and Related Health Problems, Tenth Revision (ICD-10). Four groups of suicide methods were considered in the analyses – hanging (X70), firearm (X72-X74), poisoning (X60-X69) and other methods (X71, X75-X83), which includes drowning, sharp or blunt objects, burns, explosion, fall from height, collision of motor vehicle or other moving objects, or other specified means.

Time trend analysis was performed using joinpoint regression models [19]. Segments with a minimum of four observations were considered for identifying joinpoints. The model selection criterion was based on the Bayesian Information Criterion, which enabled the selection of the simplest model with the best fit. Once the model was defined, the annual percentage change (APC) was estimated for each segment, as well as the average annual percentage change (AAPC) for the entire period, and 95% confidence intervals.

Data were analysed using Stata software, version 17.0. Trend analyses were conducted using the Joinpoint Regression Program, version 4.8.0.1 (https://surveillance.cancer.gov/joinpoint/).

### Ethics statement

The study used publicly available anonymised data from the Brazilian Mortality Information System (SIM), and hence did not require ethical approval, according to Brazilian National Ethics Commission Resolution n. 510/2016.

## Results

Between 2000 and 2022, 564,103 teenage deaths were recorded in Brazil, of which 19,873 (3.5%) were due to suicide. During the same period, 25.2% of the total number of deaths were classified as garbage codes (S1 Appendix in Supplementary Table 3).

**Fig 1:**
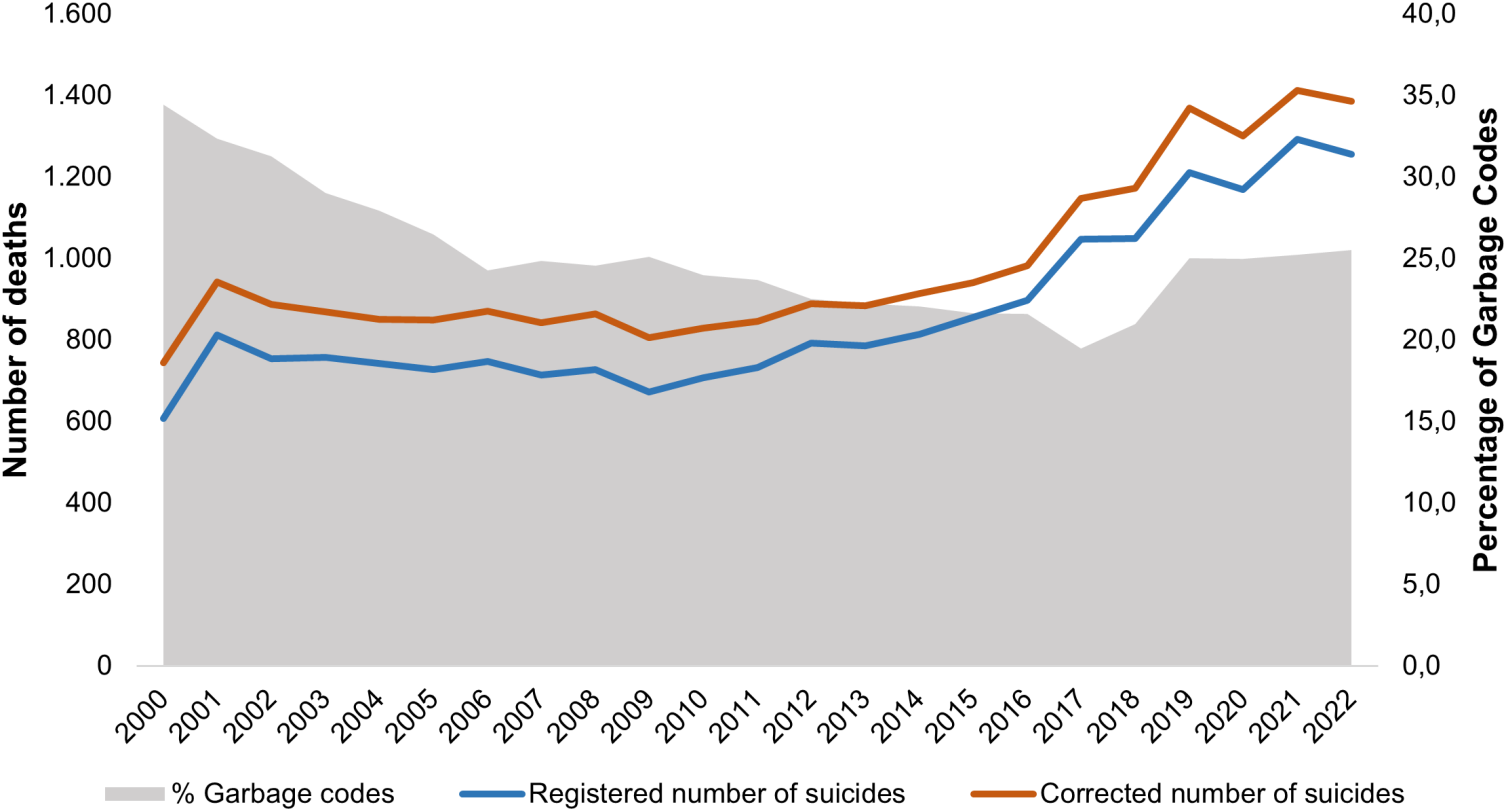
Number of suicides and percentage of deaths classified as garbage codes among adolescents. Brazil, 2000–2022.

Fig 1 illustrates the originally registered number of suicides, the estimated number of suicides after the redistribution, and the proportions of deaths classified as garbage codes among Brazilian adolescents. The registered number of suicides varied from 608 in 2000 to 1,256 in 2022, totalling 19,873 deaths (S1 Appendix in Supplementary Table 4). During the same period, the percentage of deaths classified as garbage codes declined from 34.4% in 2000 to 19.5% in 2017, followed by an increase to 25.2% in 2022 (S1 Appendix in Supplementary Table 3). Redistribution of garbage codes resulted in an average 14.3% increase in the number of adolescent suicides during the analysed period, totalling 22,591 deaths (S1 Appendix in Supplementary Table 4).

Between 2000 and 2022, the corrected suicide rates among adolescents increased by 120%, varying from 2.10 to 4.62 deaths per 100,000 adolescents (Table 1). The highest percentage increase occurred in the 10–14 age group (140%). Notably, there was a 176% increase in suicide rates among females in this age group, varying from 0.61 in 2000 to 1.68 deaths per 100,000 girls in 2022.

**Table 1:** Corrected adolescent suicide rates (per 100 thousand), by sex and age group. Brazil, 2000–2022.

	Corrected suicide rates								
	10 to 19			10 to 14			15 to 19		
Year	Total	Male	Female	Total	Male	Female	Total	Male	Female
2000	2.10	2.68	1.52	0.65	0.70	0.61	3.53	4.65	2.41
2001	2.67	3.48	1.86	0.83	0.84	0.82	4.47	6.07	2.88
2002	2.52	3.28	1.76	0.76	0.78	0.74	4.23	5.72	2.74
2003	2.47	3.25	1.69	0.70	0.78	0.62	4.20	5.68	2.73
2004	2.43	3.17	1.70	0.73	0.65	0.80	4.10	5.64	2.57
2005	2.43	3.12	1.74	0.74	0.73	0.76	4.10	5.51	2.70
2006	2.50	3.32	1.68	0.82	0.85	0.80	4.18	5.80	2.56
2007	2.43	3.28	1.58	0.83	0.95	0.71	4.03	5.63	2.43
2008	2.50	3.25	1.73	0.74	0.74	0.74	4.26	5.79	2.72
2009	2.33	3.31	1.34	0.77	0.80	0.73	3.90	5.86	1.95
2010	2.41	3.33	1.47	0.72	0.79	0.65	4.08	5.89	2.28
2011	2.46	3.26	1.66	0.76	0.88	0.64	4.15	5.64	2.66
2012	2.61	3.58	1.62	0.81	1.03	0.58	4.37	6.11	2.62
2013	2.62	3.64	1.57	0.86	0.99	0.74	4.30	6.23	2.36
2014	2.73	3.74	1.70	1.00	1.13	0.87	4.37	6.25	2.47
2015	2.85	3.89	1.77	0.93	1.05	0.81	4.64	6.58	2.67
2016	3.01	4.17	1.80	1.02	1.06	0.97	4.85	7.09	2.56
2017	3.56	4.68	2.41	1.23	1.25	1.21	5.73	7.89	3.51
2018	3.70	4.84	2.50	1.23	1.32	1.14	5.98	8.13	3.76
2019	4.39	6.05	2.66	1.42	1.33	1.52	7.15	10.46	3.72
2020	4.23	5.68	2.72	1.30	1.24	1.37	6.96	9.84	3.97
2021	4.66	5.84	3.42	1.69	1.55	1.84	7.43	9.87	4.89
2022	4.62	5.95	3.23	1.57	1.47	1.68	7.51	10.20	4.71

Male suicide rates in the 15–19 age group were, on average, 2.3 times higher than female rates (Table 1). In contrast, there was a progressive reduction in the male-to-female rate ratio in the 10 to 14 age group from 2012 to 2022, decreasing from 1.76 to 0.88.

Trend analysis of adolescent suicide mortality revealed increasing rates in both sexes and age groups (Table 2). The trends of all analysed groups presented at least one joinpoint, ranging between 2009 and 2014.

**Table 2:** Adolescent suicide mortality trends in Brazil, by sex and age group. Brazil, 2000–2022.

Gender	Age group	Time period	APC	(95% CI)	Trend	AAPC	(95% CI)	Overall trend
Both	10 to 19	2000–2013	0.43	(−0.53; 1.40)	− Stable	3.35³	(2.56; 4.14)	↑ Increasing
		2013–2022	7.72³	(6.17; 9.29)	↑ Increasing			
	10 to 14	2000–2011	0.23	(−1.40; 1.88)	− Stable	3.69³	(2.65; 4.73)	↑ Increasing
		2011–2022	7.27³	(5.79; 8.76)	↑ Increasing			
	15 to 19	2000–2013	0.22	(−0.74; 1.19)	− Stable	3.10³	(2.31; 3.90)	↑ Increasing
		2013–2022	7.42³	(5.86; 9.00)	↑ Increasing			
Male	10 to 19	2000–2012	0.72	(−0.48; 1.94)	− Stable	3.28³	(2.42; 4.15)	↑ Increasing
		2012–2022	6.44³	(5.00; 7.89)	↑ Increasing			
	10 to 14	2000–2009	1.04	(−1.51; 3.66)	− Stable	3.26³	(2.02; 4.51)	↑ Increasing
		2009–2022	4.82³	(3.49; 6.17)	↑ Increasing			
	15 to 19	2000–2013	0.80	(−0.35; 1.96)	− Stable	3.22³	(2.29; 4.16)	↑ Increasing
		2013–2022	6.83³	(5.02; 8.68)	↑ Increasing			
Female	10 to 19	2000–2013	-0.90	(−2.16; 0.37)	− Stable	3.16³	(2.11; 4.22)	↑ Increasing
		2013–2022	9.32³	(7.21; 11.48)	↑ Increasing			
	10 to 14	2000–2012	-0.97	(−2.94; 1.04)	− Stable	4.13³	(2.70; 5.57)	↑ Increasing
		2012–2022	10.59³	(8.21; 13.03)	↑ Increasing			
	15 to 19	2000–2014	-0.74	(−1.99; 0.54)	− Stable	2.87³	(1.67; 4.07)	↑ Increasing
		2014–2022	9.48³	(6.65; 12.39)	↑ Increasing			

¹ p-value <0.05; ² p-value<0.01; ³ p-value<0.001.

APC: Annual Percent Change; AAPC: Average Annual Percent Change.

Trend segments were defined using the Joinpoint Regression Program. Overlapping years represent turning points (joinpoints) where statistically significant changes in trend occurred and are included as the end of one segment and the beginning of the next. Each year is only counted once in the overall analysis.

**Fig 2:**
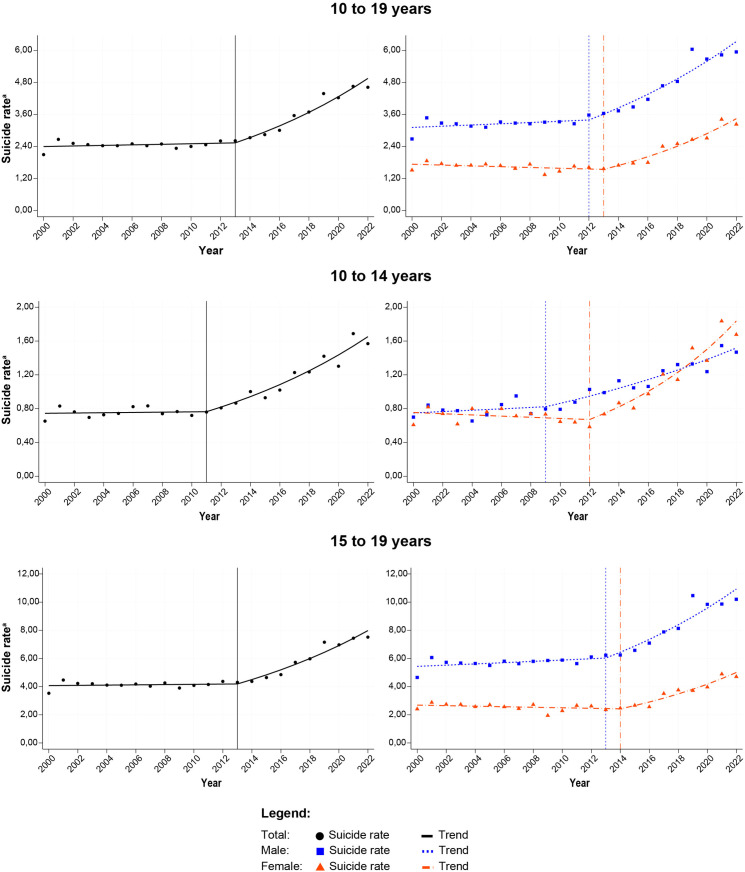
Trends in adolescent suicide rates in Brazil, by sex and age group. Brazil, 2000–2022.

On average, suicide rates increased by 3.35% per year, remaining stable between 2000 and 2013, followed by a sharp increase (APC: 7.72%; 95% CI: 6.17; 9.29). A pronounced upward trend in suicide rates among 10–14-year-old girls was observed from 2012 onwards (APC: 10.59%; 95% CI 8.21; 13.03), surpassing male rates after 2018. Among boys in the same age group, the annual increase was 4.82% (95% CI: 3.49; 6.17). Among 15–19-year-olds, suicide rates were stable from 2000–2014, followed by a sharp increasing trend (APC: 7.42%; 95% CI:5.86; 9.00) (Table 2, Fig 2).

Hanging was the most frequent method (65.9% of total deaths), followed by poisoning (15.7%) and firearms (10.8%) (S1 Appendix in Supplementary Table 5), with marked differences between sexes. Among males, hanging accounted for 70.5% of deaths, followed by firearms (12.3%), while among females, hanging accounted for 52.7% of deaths, followed by poisoning (30.8%) (S1 Appendix in Supplementary Table 5).

An increase in death rates by hanging was observed throughout the entire period, with a more pronounced trend from 2010 onwards (APC: 8.19%; 95% CI: 6.88; 9.50). Among females, the rates remained stable until 2009, followed by a strong upward trend (APC: 10.81; 95% CI: 9.21; 12.43), while among males, rates increased throughout the whole period, with an average annual growth of 5.11% (95% CI: 3.99; 6.25) (Table 3). Deaths by hanging also increased in both age groups, with an average annual growth of 5.22% (95% CI: 3.77; 6.70) for the 10–14 age group and 5.52% (95% CI: 4.39; 6.67) for young people aged 15–19 (Table 3).

**Table 3:** Adolescent suicide mortality trends in Brazil, by sex and suicide method. Brazil, 2000–2022.

		Method	Time period	APC	[95%CI]	Trend	AAPC	[95%CI]	Trend
Total		Hanging	2000–2010	2.35¹	(0.20; 4.55)	↑ Increasing	5.49³	(4.35; 6.65)	↑ Increasing
			2010–2022	8.19³	(6.88; 9.50)	↑ Increasing			
		Firearm	2000–2011	−9.83³	(−11.49; −8.14)	↓ Decreasing	−3.64³	(−4.98; −2.28)	↓ Decreasing
			2011–2022	2.97¹	(0.56; 5.43)	↑ Increasing			
		Poisoning	2000–2008	2.10	(−1.11; 5.40)	− Stable	0.11	(−2.25; 2.53)	− Stable
			2008–2015	−8.49²	(−13.57; −3.11)	↓ Decreasing			
			2015–2022	7.09²	(2.33; 12.08)	↑ Increasing			
		Other	2000–2016	1.26	(−0.04; 2.58)	− Stable	3.45³	(1.90; 5.02)	↑ Increasing
			2016–2022	9.52³	(4.36; 14.94)	↑ Increasing			
Sex	Male	Hanging	2000–2011	3.07²	(1.19; 4.98)	↑ Increasing	5.11³	(3.99; 6.25)	↑ Increasing
			2011–2022	7.20³	(5.71; 8.71)	↑ Increasing			
		Firearm	2000–2012	−8.77³	(−10.55; −6.97)	↓ Decreasing	−3.55³	(−5.15; −1.91)	↓ Decreasing
			2012–2022	3.12	(−0.11; 6.46)	− Stable			
		Poisoning	2000–2022	−0.10	(−1.28; 1.08)	− Stable	−0.10	(−1.28; 1.08)	− Stable
		Other	2000–2022	3.01³	(1.89; 4.15)	↑ Increasing	3.01³	(1.89; 4.15)	↑ Increasing
	Female	Hanging	2000–2009	0.12	(−3.30; 3.66)	− Stable	6.30³	(4.67; 7.97)	↑ Increasing
			2009–2022	10.81³	(9.21; 12.43)	↑ Increasing			
		Firearm	2000–2011	−11.79³	(−14.22; −9.30)	↓ Decreasing	−3.61³	(−5.66; −1.51)	↓ Decreasing
			2011–2022	5.34²	(1.52; 9.29)	↑ Increasing			
		Poisoning	2000–2008	1.17	(−2.58; 5.07)	− Stable	−0.39	(−3.35; 2.66)	− Stable
			2008–2015	−10.65²	(−16.98; −3.84)	↓ Decreasing			
			2015–2022	9.10²	(2.94; 15.63)	↑ Increasing			
		Other	2000–2017	0.78	(−0.87; 2.46)	− Stable	3.74³	(1.50; 6.03)	↑ Increasing
			2017–2022	14.45²	(4.99; 24.76)	↑ Increasing			
Age group	10 to 14	Hanging	2000–2010	1.00	(−1.69; 3.76)	− Stable	5.22³	(3.77; 6.70)	↑ Increasing
			2010–2022	8.88³	(7.19; 10.60)	↑ Increasing			
		Firearm	2000–2010	−8.94²	(−14.07; −3.51)	↓ Decreasing	−1.45	(−4.95; 2.17)	− Stable
			2010–2022	5.26	(−0.08; 10.88)	− Stable			
		Poisoning	2000–2022	−1.14	(−3.08; 0.83)	− Stable	−1.14	(−3.08; 0.83)	− Stable
		Other	2000–2022	5.47³	(3.33; 7.64)	↑ Increasing	5.47³	(3.33; 7.64)	↑ Increasing
	15 to 19	Hanging	2000–2011	3.04²	(1.14; 4.98)	↑ Increasing	5.52³	(4.39; 6.67)	↑ Increasing
			2011–2022	8.06³	(6.57; 9.57)	↑ Increasing			
		Firearm	2000–2012	−9.50³	(−11.12; −7.86)	↓ Decreasing	−3.98³	(−5.49; −2.45)	↓ Decreasing
			2012–2022	3.09¹	(0.00; 6.28)	↑ Increasing			
		Poisoning	2000–2008	1.31	(−1.81; 4.54)	− Stable	0.10	(−2.18; 2.44)	− Stable
			2008–2016	−7.73²	(−11.81; −3.45)	↓ Decreasing			
			2016–2022	9.81²	(3.87; 16.10)	↑ Increasing			
		Other	2000–2016	0.80	(−0.68; 2.31)	− Stable	2.95²	(1.16; 4.77)	↑ Increasing
			2016–2022	8.90²	(2.92; 15.23)	↑ Increasing			

¹ p-value <0.05; ² p-value<0.01; ³ p-value<0.001.

APC: Annual Percent Change; AAPC: Average Annual Percent Change.

Trend segments were defined using the Joinpoint Regression Program. Overlapping years represent turning points (joinpoints) where statistically significant changes in trend occurred and are included as the end of one segment and the beginning of the next. Each year is only counted once in the overall analysis.

Firearm deaths decreased in the first half of the analysed period (APC: −9.83%; 95% CI: −11.49; −8.14), with an inversion of the trend after 2011 (APC: 2.97%; 95% CI: 0.56; 5.43) (Table 3), and a notable increase in death rates from 2017 onwards. From 2011 to 2017, firearm suicide rates increased by 1.6%, while from 2017 to 2022, they increased by 43.5%. Both sexes and age groups showed changes in firearm suicide trends after 2010 (Table 3). Among boys, the decline in firearm suicides stopped after 2012, and rates became stable, while among girls, the decreasing trend reversed after 2011, with an annual rise in firearm suicide rates of 5.3% (95% CI: 1.52; 9.29). For adolescents aged 10–14, there was a decline in rates until 2010, followed by an estimated annual growth of 5.56% (95% CI: −0.08; 10.88), although not statistically significant, while in the 15–19 age group, there was a reduction until 2013, followed by an annual growth of 3.09% (95% CI: 0.00; 6.28) (Table 3).

Poisoning deaths remained stable among males throughout the entire period. Female suicide rates by poisoning were higher than male rates, with stability from 2000 to 2008, a decreasing trend until 2015 (APC: −10.65%; 95% CI: −16.98; −3.84), followed by an upward trend (APC: 9.10%; 95% CI: 2.94; 15.63) (Table 3). Suicide rates by poisoning also remained stable among 10–14-year-olds, while the 15−19 age group exhibited two turning points. Between 2000 and 2008, poisoning rates among 15–19-year-olds remained stable, followed by a decline from 2008 to 2016 (APC: −7.73%; 95% CI: −11.81; −3.45), and subsequent growth (APC: 9.81%; 95% CI: 3.87; 16.10) (Table 3).

Deaths by other means of aggression showed a consistent upward trend among males (APC: 3.01%; 95% CI: 1.89; 4.15). Among females, the rates remained stable from 2000 to 2017, followed by a sharp growth (APC: 14.45%; 95% CI: 4.99; 24.76). Suicide rates by other means of aggression also showed increasing trends in both age groups. Among 10–14-year-olds, there was an annual increase of 5.47% (95% CI: 3.33; 7.64), while among 15–19-year-olds, there was stability between 2000 and 2016, followed by an annual growth of 8.90% (95% CI: 2.92; 15.23) (Table 3).

## Discussion

The study revealed a concerning scenario regarding adolescent suicides in Brazil, with rates increasing across all sex and age subgroups and nearly all suicide methods analysed. Although the redistribution of deaths classified as garbage codes did not change the overall trends, it resulted in an average increase of 14.3% in the estimated suicide rates, emphasising the need for more accurate reporting and classification of deaths.

These findings contrast with global trends, which indicate a decline in adolescent suicides [3,20,21]. This global decline has been attributed to various interventions, such as restricted access to means of self-harm (e.g. firearms and pesticides) and expanded access to antidepressants [20,21].

In Brazil, however, persistent increases in adolescent suicide rates likely reflect local and regional challenges, and may be influenced by factors such as economic and social inequalities, and rising rates of alcohol and substance abuse [20–22]. These disparities underscore the need for interventions tailored to specific risk factors faced by Brazilian adolescents.

Adolescence is a key stage of psychosocial development, characterised by impulsivity, experimentation, risk-taking, identity formation, and major changes in interpersonal relationships, with emotional separation from parents, immersion in peer activities, and development of romantic relationships [23,24], which can increase exposure to specific risk and precipitating factors for suicidal thoughts and behaviours [25,26].

Compared to adults, adolescents are more likely to make impulsive suicide attempts with less lethal methods [26,27], often in response to acute stressors [25], and are less likely to have a diagnosed psychiatric condition or a history of mental health treatment [26,27].

In particular, interpersonal difficulties such as family conflicts, romantic relationship problems, and peer rejection are frequently cited as precipitants for adolescent suicide attempts [26–29]. These issues may also contribute to a reduced sense of belonging [30], feelings of loneliness and anxiety [29], and poor coping skills [28–30].

Gender differences in suicidal behaviours are well-documented, with males exhibiting higher suicide completion rates and females displaying higher rates of suicidal ideation and attempts [31,32]. However, this study highlights an accelerated rise in suicide rates among girls aged 10–14. This is consistent with international data showing a narrowing gender gap in adolescent suicides [21,33]. This shift may partly reflect changes in the choice of means, with females increasingly using more lethal methods such as hanging, which has traditionally been more common among males [34].

Socially constructed gender roles may influence these dynamics. Among males, traits such as impulsivity and aggressiveness [35,36], and higher rates of substance abuse [3,37] may increase the likelihood of using violent methods [35,36,38]. Conversely, females are more likely to have a history of previous attempts and to use non-violent methods, such as drug overdose [35,36,38]. However, as more lethal methods are employed, gender differences tend to diminish [31]. Therefore, the increasing use of more violent means by females underscores the need for prevention strategies that address this evolving trend.

Suicide methods play a critical role in lethality and intervention opportunities. This study found a significant rise in firearm-related suicides, particularly after 2016, coinciding with policy changes that loosened regulations and increased availability of firearms in Brazil [39,40]. Firearms are among the most lethal methods, and increased accessibility may disproportionately impact adolescents, especially in the context of impulsive suicide attempts [41]. These findings echo global research showing a threefold increase in suicide risk with greater access to firearms [42].

Evidence suggests that Brazil’s 2003 Disarmament Statute contributed to a reduction in firearm-related suicides, even though it did not change overall suicide trends, possibly due to method substitution [43]. However, regulatory relaxations starting in 2016 have contributed to increased firearm availability [39,40], potentially reversing earlier gains and driving the rise in firearm-related suicides observed in this study. Given the lethality of firearms and their role in impulsive suicides, restricting access remains an essential preventive strategy [42,44].

The predominance of deaths by hanging also reflects a concerning trend, particularly among adolescent girls. Hanging is the most common method of suicide globally due to its widespread availability and the limited possibility of restricting access [34,45]. However, evidence suggests that prevention efforts targeting adolescents at earlier stages, such as psychiatric care and crisis intervention, can contribute to preventing these deaths, especially among females [45,46].

Suicides by poisoning have also risen significantly among female adolescents since 2015, reflecting a troubling pattern of medication misuse. Data from Brazil’s Notifiable Diseases Information System (SINAN) indicate that between 2010 and 2022, nearly 80% of adolescent suicide attempts by poisoning involved medications [47], with psychotropic drugs and analgesics, especially paracetamol, being the most common agents [48–50].

While psychotropic drugs can be protective against suicide when used under supervision, their unsupervised use poses significant risks, particularly among vulnerable adolescents [51]. Preventive measures, such as limiting the availability of over-the-counter medications and restricting pack sizes [52], closely monitoring prescriptions, and prioritising substances with lower levels of toxicity for at-risk adolescents, are essential to reduce these events [51]. Additionally, public awareness campaigns targeting parents and caregivers can help mitigate the risks associated with easy access to medications at home.

The increasing rates of adolescent suicides suggest a deterioration in the mental health of Brazilian adolescents, which is supported by results from the National School Health Survey (PeNSE) [53]. In 2019, 17.7% of students aged 13–17 reported negative self-rated mental health, with significantly higher rates among girls [53]. Over 50% reported to feel excessively worried about daily activities, 31% reported to feel sad, and 21% felt hopeless most of the time, with worse results among teenage girls [53].

Recent studies have also pointed to problematic social media use as a potential risk factor for suicidal thoughts and behaviours, particularly among girls [54,55]. Excessive social media use has been associated with feelings of loneliness, low self-esteem, and distorted body image [54], all of which may contribute to suicidal behaviours [55]. Coupled with the increasing prevalence of bullying and body image dissatisfaction among Brazilian adolescents [56], the role of social media in shaping adolescent mental health warrants further investigation.

Economic factors may also play a significant role. The sharp rise in suicide rates among 15–19-year-olds after 2014 coincides with Brazil’s economic recession, which led to increased rates of poverty and unemployment, as well as budget cuts in social, health and education programmes [57,58]. Literature has shown associations between increased inequality and unemployment and depression, suicidal ideation and attempts, and suicide deaths in both adults and adolescents [59–61]. Previous studies suggest that parental job loss and financial hardship may lead to increased family distress and conflict, as well as heightened feelings of burdensomeness and hopelessness, especially among older adolescents, which can lead to increased risks of suicidal behaviours [59,60,62].

Lastly, although the present study did not aim to assess the impact of the COVID-19 pandemic, it is important to acknowledge that the pandemic may have long-term implications for adolescents’ mental health and suicidal behaviours. Even though studies on the effects of the pandemic on suicide deaths have yielded mixed results [63], evidence suggests an increase in prevalence rates of suicidal ideation and attempts following the beginning of the public health emergency [64,65]. Factors such as social isolation, family conflicts, and increased online activity likely exacerbated mental health vulnerabilities, which can contribute to higher rates of suicidal behaviours over time [63]. These findings highlight the need for sustained mental health support and resilience-building strategies in the aftermath of the pandemic.

This study has limitations associated with the use of secondary data, particularly the variations in the proportion of deaths classified as undetermined intent over the years. Redistribution of GCs is a potential strategy to address this limitation. However, while this method improves trend accuracy, it introduces its own limitations, as it assumes that the distribution of GCs among target causes is consistent with predefined redistribution weights. Consequently, redistribution weights obtained from the 60 Cities Project may not be consistent with the real distribution of garbage codes in the entire country throughout the whole period. Moreover, limitations related to the coverage of death records must also be acknowledged. Failures in death registration and underreporting of suicides, particularly in regions with limited healthcare and forensic capacity, can lead to underestimation of suicide rates.

To mitigate these limitations, we combined proportional and empirical redistribution approaches, based on data from previous investigations of deaths classified as GCs, applied stratified analyses by gender, age group, and method, and used joinpoint regression models to enhance the robustness of trend estimations. Although redistribution cannot eliminate all potential biases, such as underestimation of suicide rates due to underreporting, these measures reduce the likelihood that data quality issues significantly compromise the results.

In addition, the ecological nature of the study prevents the establishment of causal relationships between suicide trends and individual or contextual risk factors, such as social media use, economic downturns, or the COVID-19 pandemic. In-depth studies are needed to clarify possible causal relationships with these events.

## Conclusion

The study’s results highlight a concerning increase in adolescent suicide rates in Brazil, with a particularly alarming rise among females and an increasing use of more lethal methods. The study offers updated insights into sex-, age- and method-specific suicide trends, emphasising the importance of addressing data quality issues to improve the accuracy of suicide estimates and inform policymaking.

The rising trends of teenage suicides underscore the need for comprehensive efforts to address the multifaceted nature of adolescent suicide and its determinants. Future research should further investigate the underlying causes driving these upward trends, with particular attention to the influence of socioeconomic factors, social media exposure, and the potential long-term effects of the COVID-19 pandemic.

Despite the complexity of factors influencing adolescent suicides, these deaths are preventable through targeted and evidence-based strategies that extend beyond individual interventions to address broader social and environmental contexts shaping adolescent suicidal behaviours [66]. Restricting access to lethal means, such as firearms and certain medications, remains critical but insufficient as a standalone measure. Therefore, policymakers must prioritise mental health promotion, strengthen psychosocial care, and ensure equitable access to high-quality mental health services for adolescents at risk.

In addition, educational campaigns in schools and communities should be implemented to raise awareness, reduce stigma, and encourage early help-seeking behaviours. A multidisciplinary and integrated approach, encompassing prevention, intervention, and postvention strategies, is essential to safeguard the mental health and well-being of Brazil’s youth.

## Supporting information

S1 Appendix Supplementary information on the redistribution methods and results. (DOCX)

S1 Fig Redistribution of deaths classified as Garbage Codes. Brazil, 2000–2022. (PDF)
