# Supplementary material for: Adolescent suicide trends in Brazil (2000–2022): An ecological analysis by sex, age, and suicide methods
Source: PLoS One. 2025 Jul 18;20(7):e0309505. doi: 10.1371/journal.pone.0309505 (PMC12273947; doi:10.1371/journal.pone.0309505)
Supplement: S1 Appendix — (DOCX) [file pone.0309505.s001.docx]

**Supplementary materials**

**Methods for the redistribution of garbage codes:**

To address the misclassification of suicide deaths, a redistribution procedure for garbage codes (GCs) was implemented, adapting the methodology proposed by Soares Filho et al. and compiled by Teixeira in the R package RedGCSIM. The original approach was modified to allow for stratification by suicide method and to improve the accuracy of redistribution process.

The redistribution process consisted of four main steps. First, ICD-10 garbage code groups were identified and mapped to target cause-of-death groups. The classification scheme considered both natural and external causes, incorporating stratifications based on the injury mechanism for self-harm, aggression, or accidental injuries. The predefined injury mechanisms included transport-related injuries (including motor vehicle crashes), fall from height, impact of moving object, poisoning, hanging, drowning, sharp object, firearms and a group of other specified mechanisms (Supplementary table 1). In the second step, missing information on sex and age was proportionally redistributed within each year and ICD-10 code group.

The third step involved the redistribution of deaths initially classified as GCs. Deaths with unspecified injury mechanisms were proportionally redistributed to specified injury mechanisms. Deaths with undetermined intent with a specified mechanism were redistributed to corresponding categories of intentional or unintentional injuries. Deaths by other unspecified injuries (‘GC _injuries’) were redistributed proportionally across all injuries with specified intent and mechanism.

Additionally, garbage codes X59 (Exposure to unspecified factor), Y34 (Unspecified event, undetermined intent) and unspecified pneumonia (GC _pneumonia) and other gargage codes (GC _all) were redistributed using predefined redistribution weights derived from the 60 Cities Project, which provided empirically based estimates of the likely causes of death based on post-mortem investigations.

An additional refinement was applied to the redistribution of deaths classified under the ‘GC _all’ ICD-10 garbage code group, which includes ICD-10 codes that could be redistributed to both natural and external causes. While the original methodology used a proportional redistribution approach across all cause-of-death categories, further analysis indicated that this approach could lead to a higher allocation of violent deaths, particularly homicides, among younger age groups. To better align the redistribution with observed mortality patterns, the redistribution of ‘GC _all’ was adjusted by applying predefined weights from the 60 Cities Project, stratified by age group. This refinement enhanced the accuracy of the redistribution process, ensuring that redistributed deaths closely reflected the findings of empirical investigations, while maintaining consistency with the methodological framework.

The final corrected suicide rates were estimated using the redistributed data, stratified by sex, age group, and method. Supplementary Table 1 provides the full list of ICD-10 codes and their respective redistribution targets. Supplementary Table 2 details the predefined redistribution weights applied to each group of garbage codes and their respective target groups. The redistribution workflow is illustrated in Supplementary Figure 1.

**Supplementary table 1.** List of ICD-10 code groups*.*

| **ICD-10 Code** | **ICD-10 Codes:** | **Redistribution method** | **Target groups** |
| --- | --- | --- | --- |
| **Groups** |  |  |  |
| **Natural causes of death** | | | |
| Natural causes (NAT nat_cause) | A00-A39, A42-A46, A481, A482, A484, A488, A492-A499, A50-A58, A60-A70, A748, A749, A75-A99, B00-B06, B08-B29, B33-B34, B37-B83, B86, B89-B93, B941-B949, B95-B99, D00-D64, D66, D67, D680-D689, D69-D14, E160-E169, E20-E46, E51-E63, E640, E642-E649, E65-E86, E870, E871, E874-E878, E88-E90, F00-F05, F060-F062, F065-F069, F070, F071, F10-F16, F18-F29, F50, G00-G31, G35-G41, G45-G46, G473, G61, G70-G90, G910-G912, G918, G919, G92-G98, H050, H051, H70, I00-I45, I47-I49, I510-I516, I518, I519, I51-I89, I952, I97-I99, J00-J14, J150-J158, J16, J17, J20-J70, J82-J95, J961, J980-J982, J984-J989, J99, K20-K29, K31-K93, L00-L14, L51, L559, L589, L88, L89, M00-M09, M121. M30-M42, M430, M431, M490, M491, M650, M710, M711, M726, M730, M731, M80-M82, M86, M870, M871, M88, M890, M895, M899, N00-N31, N320-N324, N34, N36, N390-N392, N399, N40, N41, N44, N45, N49, N60, N70-N90, N92-N94, N950, N96, N98-N99, O00-O99, P00-P96, Q00-Q99, R502, R73, R780-R785, R95, U04, U06, U82-U85 | - | - |
| **Road traffic injuries** | | | |
| Pedestrian  (INJ road_pedestr) | V01-V09 | - | - |
| Cyclist  (INJ road_cyclist) | V10-V19 | - | - |
| Motor vehicle  (INJ road_motor) | V20-V79, V81-V88 | - | - |
| Non-motor vehicle  (INJ road_non_motor) | V80 | - | - |
| **Other transport injuries** | | | |
| Other transport injuries  (INJ transp_other) | V90-V99, Y85 | - | - |
| **Accidental fall from height** | | | **-** |
| Fall from height  (INJ acd_fall_h) | W00-W19 | - | - |
| **Other accidents** | | | |
| Poisoning  (INJ acd_poison) | X40-X49 | - | - |
| Hanging  (INJ acd_hang) | W76 | - | - |
| Drowning  (INJ acd_drown) | W65-W74 | - | - |
| Sharp objects  (INJ acd_sharp) | W25-W27 | - | - |
| Firearm  (INJ acd_firearm) | W32-W34 | - | - |

(Continues...)

(Continuation...)

| **ICD-10 Code** | **ICD-10 Codes:** | **Redistribution method** | **Target groups** |
| --- | --- | --- | --- |
| **Groups** |  |  |  |
| **Other accidents** | | | |
| Struck, crushed, jammed or pinched by moving objects  (INJ acd_fall_m) | W20-W23 | - | - |
| Other accidental injuries  (INJ acd_other) | W24, W28-W31, W35-W64, W75, W77-W99, X00-X39, X50-X58 | - | - |
| **Other external causes** | | | |
| Other external causes  (INJ ce_other) | L550-L558, L563, L568, L580, L581, Y36-Y84, Y88, Y890, Y891 | - | - |
| **Suicides** | | | |
| Poisoning  (INJ sui_poison) | X60-X69 | - | - |
| Hanging, strangulation or suffocation  (INJ sui_hang) | X70 | - | - |
| Drowning  (INJ sui_drown) | X71 | - | - |
| Firearm  (INJ sui_firearm) | X72-X74 | - | - |
| Sharp object  (INJ sui_sharp) | X78 | - | - |
| Fall from height (INJ sui_fall_h) | X80 | - | - |
| Jumping or lying before moving object  (INJ sui_fall_m) | X81 | - | - |
| Crashing of motor vehicle (INJ sui_motor) | X82 | - | - |
| Other methods  (INJ sui_other) | X75-X77, X79, X83 | - | - |
| **Homicides** | | | |
| Poisoning  (INJ agr_poison) | X85, X87-X90 | - | - |
| Hanging, strangulation or suffocation  (INJ agr_hang) | X91 | - | - |
| Drowning  (INJ agr_drown) | X92 | - | - |

(Continues...)

(Continuation...)

| **ICD-10 Code** | **ICD-10 Codes:** | **Redistribution method** | **Target groups** |
| --- | --- | --- | --- |
| **Groups** |  |  |  |
| **Homicides** | | | |
| Firearm  (INJ agr_firearm) | X93-X95, Y350 | - | - |
| Sharp object  (INJ agr_sharp) | X99, Y354 | - | - |
| Fall from height  (INJ agr_fall_h) | Y01 | - | - |
| Pushed or placed before moving object  (INJ agr_fall_m) | Y02 | - | - |
| Crashing of motor vehicle  (INJ agr_motor) | Y03 | - | - |
| Other methods  (INJ agr_other) | X86, X96-X98, Y00, Y04-Y08, Y351-Y353, Y355, Y356 | - | - |
| **Garbage code groups** | | | |
| Other garbage codes  (GC _all) | A40-A41, A480, A490, A491, A59, A71, A740, B07, B30, B35-B36, B85, B87, B88, B940, D65, E15, E50, E641, E872, E873, F063, F064, F078, F079, F09, F17, F30-F48, F51-F99, G32, F43, G440-G442, G444, G448, G470-G472, G474-G479, G50-G60, G62-G64, G99, H00-H04, H052-H059, H06-H69, H71-H95, I46, I50, I517, I950, I951, I958, I959, J80, J81, J960, J969, J983, K00-K14, K30, L20-L50, L52-L54, L560-L562, L564, L57, L59-L87, L93, L97, L980-L984, L90-L92, L94, L95, L985-L989, L99, M10, M11, M120, M122-M128, M13-M25, M432-M439, M45-M48, M492-M498, M50-M63, M651-M659, M66-M70, M712-M719, M720-M724, M728, M729, M738, M75-M79, M83-M85, M872-M879, M891-M894, M90-M99, N328, N329, N33, N35, N37, N39, N42, N43, N46-N48, N50, N51, N61-N64, N91, N951-N959, N97, R00-R49, R508, R509, R51-R57, R59-R72, R74-R77, R786-R789, R79-R94, R96-R99, U07-U49, Z00-Z99 | Predefined weights | Predefined weights |
| Unspec. pneumonia  (GC _pneumo) | J159, J18 | Predefined weights | Predefined weights |
| X59 (GC _X59) | X59 | Predefined weights | Predefined weights |
| Y34 (GC _Y34) | Y34 | Predefined weights | Predefined weights |
| Unspec. injuries  (GC _injuries) | G443, G913, R58, S00-S99, T00-T98, Y86, Y87, Y899, Y90-Y98 | Proportional | All external causes |
| Unspec. road traffic injuries  (GC road_nspe) | V89 | Proportional | Road traffic injuries |
| Unspec. suicide  (GC sui_nspe) | X84 | Proportional | Suicides |
| Unspec. homicide  (GC agr_nspe) | Y09, Y357 | Proportional | Homicides |

(Continues...)

(Continues...)

| **ICD-10 Code** | **ICD-10 Codes:** | **Redistribution method** | **Target groups** |
| --- | --- | --- | --- |
| **Groups** |  |  |  |
| **Garbage code groups** | | | |
| Undet. poisoning  (GC ind_poison) | Y10-Y19 | Proportional | Poisonings |
| Undet. hanging  (GC ind_hang) | Y20 | Proportional | Hangings |
| Undet. drowning  (GC ind_drown) | Y21 | Proportional | Drownings |
| Undet. firearm  (GC ind_firearm) | Y22-Y24 | Proportional | Firearms |
| Undet. sharp object  (GC ind_sharp) | Y28 | Proportional | Sharp objects |
| Undet. fall from height  (GC ind_fall_h) | Y30 | Proportional | Fall from height |
| Undet. struck, crushed, jammed or pinched by, jumping, lying, pushed or placed before moving object  (GC ind_fall_m) | Y31 | Proportional | Struck, crushed, jammed or pinched by, jumping, lying, pushed or placed before moving object |
| Undet. crashing of motor vehicle  (GC ind_motor) | Y32 | Proportional | Crashing of motor vehicle |
| Undet. other methods  (GC ind_other) | Y25-Y27, Y29, Y33 | Proportional | Other injuries |

**Supplementary table 2.** Predefined redistribution weights, based on the results of the 60 Cities Project. Brazil, 2017-2018*.*

| **GC  Group** | **ICD-10 Target  Group** | **Age group** | | |
| --- | --- | --- | --- | --- |
|  |  | 0 to 9 years | 10 to 59 years | 60 years or older |
| X59 | Natural causes of death | 0,00000000000000000 | 0,01228070175438600 | 0,04213483146067400 |
|  | Road traffic injuries | 0,52631578947368400 | 0,77894736842105300 | 0,25000000000000000 |
|  | Other transport injuries | 0,05263157894736800 | 0,00350877192982500 | 0,00000000000000000 |
|  | Accidental fall | 0,00000000000000000 | 0,09298245614035100 | 0,67134831460674200 |
|  | Other accidents | 0,42105263157894700 | 0,06315789473684200 | 0,02528089887640500 |
|  | Suicide | 0,00000000000000000 | 0,00526315789473700 | 0,00280898876404500 |
|  | Homicide | 0,00000000000000000 | 0,04385964912280700 | 0,00842696629213500 |
|  | Other external causes | 0,00000000000000000 | 0,00000000000000000 | 0,00000000000000000 |
| Y34 | Natural causes of death | 0,28089887640449400 | 0,05122997066125000 | 0,09051929490233400 |
|  | Road traffic injuries | 0,15730337078651700 | 0,20266305574362400 | 0,15292996665078600 |
|  | Other transport injuries | 0,00000000000000000 | 0,00225682690137700 | 0,00142925202477400 |
|  | Accidental fall | 0,07865168539325800 | 0,08350259535093700 | 0,65173892329680800 |
|  | Other accidents | 0,31460674157303400 | 0,04716768223877200 | 0,03287279656979500 |
|  | Suicide | 0,01123595505618000 | 0,04874746106973600 | 0,02191519771319700 |
|  | Homicide | 0,15730337078651700 | 0,56443240803430400 | 0,04668889947594100 |
|  | Other external causes | 0,00000000000000000 | 0,00000000000000000 | 0,00190566936636500 |
| Pneumonia | Natural causes of death | 0,94927536231884100 | 0,94410569105691100 | 0,93598382749326100 |
|  | Road traffic injuries | 0,00000000000000000 | 0,01117886178861800 | 0,00292003593890400 |
|  | Other transport injuries | 0,00000000000000000 | 0,00203252032520300 | 0,00022461814914600 |
|  | Accidental fall | 0,00000000000000000 | 0,01422764227642300 | 0,05098831985624400 |
|  | Other accidents | 0,05072463768115900 | 0,00711382113821100 | 0,00673854447439400 |
|  | Suicide | 0,00000000000000000 | 0,00813008130081300 | 0,00067385444743900 |
|  | Homicide | 0,00000000000000000 | 0,00914634146341500 | 0,00112309074573200 |
|  | Other external causes | 0,00000000000000000 | 0,00406504065040700 | 0,00134770889487900 |
| All | Natural causes of death | 0,86283185840708000 | 0,79831495735385900 | 0,95660004392708100 |
|  | Road traffic injuries | 0,01179941002949900 | 0,03193259829415400 | 0,00373380188886400 |
|  | Other transport injuries | 0,00000000000000000 | 0,00218431454129400 | 0,00030748956731800 |
|  | Accidental fall | 0,00442477876106200 | 0,01529020178905800 | 0,02833296727432500 |
|  | Other accidents | 0,10029498525073700 | 0,05762429789889700 | 0,00575444761695600 |
|  | Suicide | 0,00000000000000000 | 0,03245267318493900 | 0,00237206237645500 |
|  | Homicide | 0,01622418879056100 | 0,06032868733097600 | 0,00162530199868200 |
|  | Other external causes | 0,00442477876106200 | 0,00187226960682300 | 0,00127388535031800 |

**Supplementary table 3.** Number and percentage of deaths classified as garbage codes. Brazil, 2000-2022.

| **Year** | **Number of deaths** | | | **% Garbage codes** |
| --- | --- | --- | --- | --- |
|  | **Total** | **Suicide** | **Garbage** |  |
| 2000 | 25.558 | 608 | 8.804 | 34,4 |
| 2001 | 25.719 | 812 | 8.324 | 32,4 |
| 2002 | 26.205 | 754 | 8.192 | 31,3 |
| 2003 | 25.463 | 757 | 7.393 | 29,0 |
| 2004 | 25.021 | 743 | 6.992 | 27,9 |
| 2005 | 24.764 | 728 | 6.557 | 26,5 |
| 2006 | 24.740 | 748 | 6.005 | 24,3 |
| 2007 | 24.453 | 714 | 6.080 | 24,9 |
| 2008 | 24.664 | 728 | 6.059 | 24,6 |
| 2009 | 24.443 | 672 | 6.131 | 25,1 |
| 2010 | 24.671 | 707 | 5.915 | 24,0 |
| 2011 | 25.274 | 733 | 5.981 | 23,7 |
| 2012 | 26.979 | 792 | 6.070 | 22,5 |
| 2013 | 26.867 | 785 | 5.975 | 22,2 |
| 2014 | 27.445 | 814 | 6.051 | 22,0 |
| 2015 | 26.249 | 855 | 5.679 | 21,6 |
| 2016 | 26.665 | 897 | 5.762 | 21,6 |
| 2017 | 26.120 | 1.048 | 5.089 | 19,5 |
| 2018 | 23.465 | 1.049 | 4.923 | 21,0 |
| 2019 | 20.925 | 1.211 | 5.238 | 25,0 |
| 2020 | 20.522 | 1.169 | 5.123 | 25,0 |
| 2021 | 19.383 | 1.293 | 4.886 | 25,2 |
| 2022 | 18.508 | 1.256 | 4.724 | 25,5 |
| **Total** | **564.103** | **19.873** | **141.953** | **25,2** |

**Supplementary table 4.** Registered and redistributed number of suicides and suicide rates. Brazil, 2000-2022.

| **Group** | | **Metric** | **2000** | **2001** | **2002** | **2003** | **2004** | **2005** | **2006** | **2007** | **2008** | **2009** | **2010** | **2011** | **2012** | **2013** | **2014** | **2015** | **2016** | **2017** | **2018** | **2019** | **2020** | **2021** | **2022** |
| --- | --- | --- | --- | --- | --- | --- | --- | --- | --- | --- | --- | --- | --- | --- | --- | --- | --- | --- | --- | --- | --- | --- | --- | --- | --- |
| 10 to 19 | Total | N¹ | 608 | 812 | 754 | 757 | 743 | 728 | 748 | 714 | 728 | 672 | 707 | 733 | 792 | 785 | 814 | 855 | 897 | 1048 | 1049 | 1211 | 1169 | 1293 | 1256 |
|  |  | Rate¹ | 1,72 | 2,30 | 2,14 | 2,16 | 2,12 | 2,09 | 2,15 | 2,06 | 2,10 | 1,95 | 2,05 | 2,14 | 2,33 | 2,32 | 2,43 | 2,59 | 2,75 | 3,26 | 3,31 | 3,88 | 3,80 | 4,26 | 4,19 |
|  |  | Red. N² | 744 | 943 | 887 | 868 | 851 | 848 | 871 | 842 | 864 | 806 | 829 | 845 | 889 | 884 | 913 | 941 | 982 | 1147 | 1171 | 1370 | 1300 | 1412 | 1386 |
|  |  | Red. Rate² | 2,10 | 2,67 | 2,52 | 2,47 | 2,43 | 2,43 | 2,50 | 2,43 | 2,50 | 2,33 | 2,41 | 2,46 | 2,61 | 2,62 | 2,73 | 2,85 | 3,01 | 3,56 | 3,70 | 4,39 | 4,23 | 4,66 | 4,62 |
|  | Male | N¹ | 384 | 533 | 493 | 500 | 482 | 468 | 496 | 484 | 480 | 483 | 490 | 487 | 554 | 555 | 561 | 593 | 634 | 700 | 696 | 834 | 807 | 832 | 825 |
|  |  | Rate¹ | 2,17 | 3,02 | 2,80 | 2,85 | 2,75 | 2,68 | 2,85 | 2,79 | 2,77 | 2,78 | 2,83 | 2,82 | 3,22 | 3,25 | 3,31 | 3,53 | 3,82 | 4,27 | 4,31 | 5,24 | 5,14 | 5,37 | 5,38 |
|  |  | Red. N² | 475 | 614 | 578 | 571 | 554 | 545 | 578 | 570 | 565 | 575 | 577 | 562 | 615 | 622 | 634 | 652 | 693 | 767 | 782 | 963 | 892 | 905 | 912 |
|  |  | Red. Rate² | 2,68 | 3,48 | 3,28 | 3,25 | 3,17 | 3,12 | 3,32 | 3,28 | 3,25 | 3,31 | 3,33 | 3,26 | 3,58 | 3,64 | 3,74 | 3,89 | 4,17 | 4,68 | 4,84 | 6,05 | 5,68 | 5,84 | 5,95 |
|  | Female | N¹ | 224 | 279 | 261 | 257 | 261 | 260 | 252 | 230 | 248 | 189 | 217 | 246 | 238 | 230 | 253 | 262 | 263 | 348 | 353 | 377 | 362 | 461 | 431 |
|  |  | Rate¹ | 1,26 | 1,58 | 1,48 | 1,46 | 1,49 | 1,49 | 1,45 | 1,33 | 1,44 | 1,10 | 1,27 | 1,45 | 1,41 | 1,38 | 1,53 | 1,61 | 1,64 | 2,20 | 2,27 | 2,47 | 2,41 | 3,11 | 2,94 |
|  |  | Red. N² | 269 | 329 | 310 | 297 | 297 | 303 | 292 | 273 | 299 | 231 | 252 | 282 | 273 | 262 | 280 | 289 | 289 | 380 | 389 | 407 | 408 | 507 | 474 |
|  |  | Red. Rate² | 1,52 | 1,86 | 1,76 | 1,69 | 1,70 | 1,74 | 1,68 | 1,58 | 1,73 | 1,34 | 1,47 | 1,66 | 1,62 | 1,57 | 1,70 | 1,77 | 1,80 | 2,41 | 2,50 | 2,66 | 2,72 | 3,42 | 3,23 |
| 10 to 14 | Total | N¹ | 83 | 107 | 108 | 99 | 103 | 104 | 117 | 116 | 96 | 106 | 101 | 105 | 117 | 119 | 142 | 132 | 143 | 174 | 163 | 189 | 164 | 218 | 203 |
|  |  | Rate¹ | 0,47 | 0,61 | 0,62 | 0,57 | 0,59 | 0,60 | 0,67 | 0,67 | 0,55 | 0,61 | 0,59 | 0,62 | 0,70 | 0,72 | 0,87 | 0,83 | 0,91 | 1,12 | 1,07 | 1,26 | 1,11 | 1,49 | 1,39 |
|  |  | Red. N² | 115 | 145 | 132 | 121 | 126 | 129 | 143 | 144 | 128 | 132 | 124 | 129 | 136 | 143 | 163 | 148 | 160 | 190 | 188 | 214 | 193 | 247 | 229 |
|  |  | Red. Rate² | 0,65 | 0,83 | 0,76 | 0,70 | 0,73 | 0,74 | 0,82 | 0,83 | 0,74 | 0,77 | 0,72 | 0,76 | 0,81 | 0,86 | 1,00 | 0,93 | 1,02 | 1,23 | 1,23 | 1,42 | 1,30 | 1,69 | 1,57 |
|  | Male | N¹ | 41 | 54 | 53 | 54 | 44 | 50 | 58 | 65 | 49 | 54 | 54 | 59 | 77 | 70 | 80 | 76 | 75 | 89 | 87 | 85 | 81 | 99 | 98 |
|  |  | Rate¹ | 0,47 | 0,62 | 0,61 | 0,62 | 0,51 | 0,58 | 0,67 | 0,75 | 0,56 | 0,62 | 0,62 | 0,68 | 0,90 | 0,83 | 0,96 | 0,93 | 0,93 | 1,12 | 1,12 | 1,10 | 1,07 | 1,32 | 1,31 |
|  |  | Red. N² | 62 | 74 | 68 | 67 | 57 | 63 | 74 | 83 | 64 | 69 | 69 | 76 | 88 | 83 | 94 | 85 | 85 | 99 | 103 | 102 | 94 | 116 | 110 |
|  |  | Red. Rate² | 0,70 | 0,84 | 0,78 | 0,78 | 0,65 | 0,73 | 0,85 | 0,95 | 0,74 | 0,80 | 0,79 | 0,88 | 1,03 | 0,99 | 1,13 | 1,05 | 1,06 | 1,25 | 1,32 | 1,33 | 1,24 | 1,55 | 1,47 |
|  | Female | N¹ | 42 | 53 | 55 | 45 | 59 | 54 | 59 | 51 | 47 | 52 | 47 | 46 | 40 | 49 | 62 | 56 | 68 | 85 | 76 | 104 | 83 | 119 | 105 |
|  |  | Rate¹ | 0,48 | 0,61 | 0,63 | 0,52 | 0,68 | 0,62 | 0,68 | 0,59 | 0,55 | 0,61 | 0,55 | 0,55 | 0,48 | 0,60 | 0,78 | 0,72 | 0,88 | 1,12 | 1,02 | 1,42 | 1,15 | 1,66 | 1,48 |
|  |  | Red. N² | 53 | 71 | 64 | 53 | 69 | 66 | 69 | 62 | 64 | 63 | 55 | 54 | 48 | 60 | 69 | 63 | 75 | 91 | 85 | 111 | 99 | 131 | 119 |
|  |  | Red. Rate² | 0,61 | 0,82 | 0,74 | 0,62 | 0,80 | 0,76 | 0,80 | 0,71 | 0,74 | 0,73 | 0,65 | 0,64 | 0,58 | 0,74 | 0,87 | 0,81 | 0,97 | 1,21 | 1,14 | 1,52 | 1,37 | 1,84 | 1,68 |
| 15 to 19 | Total | N¹ | 525 | 705 | 646 | 658 | 640 | 624 | 631 | 598 | 632 | 566 | 606 | 628 | 675 | 666 | 672 | 723 | 754 | 874 | 886 | 1022 | 1005 | 1075 | 1053 |
|  |  | Rate¹ | 2,94 | 3,95 | 3,62 | 3,70 | 3,62 | 3,56 | 3,63 | 3,45 | 3,66 | 3,28 | 3,51 | 3,64 | 3,92 | 3,87 | 3,91 | 4,23 | 4,45 | 5,23 | 5,39 | 6,32 | 6,32 | 6,86 | 6,84 |
|  |  | Red. N² | 629 | 798 | 755 | 748 | 725 | 719 | 728 | 698 | 736 | 674 | 705 | 715 | 753 | 741 | 751 | 793 | 821 | 957 | 983 | 1156 | 1107 | 1164 | 1157 |
|  |  | Red. Rate² | 3,53 | 4,47 | 4,23 | 4,20 | 4,10 | 4,10 | 4,18 | 4,03 | 4,26 | 3,90 | 4,08 | 4,15 | 4,37 | 4,30 | 4,37 | 4,64 | 4,85 | 5,73 | 5,98 | 7,15 | 6,96 | 7,43 | 7,51 |
|  | Male | N¹ | 343 | 479 | 440 | 446 | 438 | 418 | 438 | 419 | 431 | 429 | 436 | 428 | 477 | 485 | 481 | 517 | 559 | 611 | 609 | 749 | 726 | 733 | 727 |
|  |  | Rate¹ | 3,86 | 5,38 | 4,94 | 5,03 | 4,97 | 4,78 | 5,04 | 4,84 | 4,99 | 4,97 | 5,05 | 4,96 | 5,52 | 5,61 | 5,56 | 6,00 | 6,53 | 7,21 | 7,29 | 9,11 | 8,96 | 9,17 | 9,24 |
|  |  | Red. N² | 413 | 540 | 509 | 504 | 498 | 482 | 504 | 487 | 500 | 505 | 508 | 487 | 528 | 539 | 540 | 567 | 607 | 668 | 679 | 861 | 798 | 789 | 802 |
|  |  | Red. Rate² | 4,65 | 6,07 | 5,72 | 5,68 | 5,64 | 5,51 | 5,80 | 5,63 | 5,79 | 5,86 | 5,89 | 5,64 | 6,11 | 6,23 | 6,25 | 6,58 | 7,09 | 7,89 | 8,13 | 10,46 | 9,84 | 9,87 | 10,20 |
|  | Female | N¹ | 182 | 226 | 206 | 212 | 202 | 206 | 193 | 179 | 201 | 137 | 170 | 200 | 198 | 181 | 191 | 206 | 195 | 263 | 277 | 273 | 279 | 342 | 326 |
|  |  | Rate¹ | 2,03 | 2,52 | 2,30 | 2,38 | 2,28 | 2,35 | 2,21 | 2,07 | 2,33 | 1,59 | 1,97 | 2,32 | 2,31 | 2,11 | 2,24 | 2,43 | 2,33 | 3,19 | 3,42 | 3,44 | 3,58 | 4,46 | 4,33 |
|  |  | Red. N² | 216 | 258 | 246 | 244 | 228 | 237 | 223 | 211 | 236 | 168 | 197 | 229 | 225 | 202 | 211 | 226 | 214 | 289 | 304 | 295 | 309 | 375 | 355 |
|  |  | Red. Rate² | 2,41 | 2,88 | 2,74 | 2,73 | 2,57 | 2,70 | 2,56 | 2,43 | 2,72 | 1,95 | 2,28 | 2,66 | 2,62 | 2,36 | 2,47 | 2,67 | 2,56 | 3,51 | 3,76 | 3,72 | 3,97 | 4,89 | 4,71 |

¹ Observed values. ² Redistributed values.

**Supplementary table 5.** Absolute and proportional distribution of suicide deaths by method, stratified by sex. Brazil, 2000-2022.

| **Sex** | **Year** | **Hanging** | | **Poisoning** | | **Firearm** | | **Other** | | **Total** | |
| --- | --- | --- | --- | --- | --- | --- | --- | --- | --- | --- | --- |
|  |  | **N** | **%** | **N** | **%** | **N** | **%** | **N** | **%** | **N** | **%** |
| **Both** | 2000 | 330,4 | 44,4 | 153,6 | 20,7 | 199,0 | 26,8 | 60,7 | 8,2 | 743,7 | 100,0 |
|  | 2001 | 456,4 | 48,4 | 200,2 | 21,2 | 217,4 | 23,1 | 69,1 | 7,3 | 943,0 | 100,0 |
|  | 2002 | 436,8 | 49,2 | 200,3 | 22,6 | 185,0 | 20,8 | 65,2 | 7,3 | 887,3 | 100,0 |
|  | 2003 | 467,5 | 53,8 | 170,4 | 19,6 | 181,6 | 20,9 | 48,9 | 5,6 | 868,3 | 100,0 |
|  | 2004 | 484,0 | 56,9 | 165,5 | 19,4 | 143,1 | 16,8 | 58,7 | 6,9 | 851,2 | 100,0 |
|  | 2005 | 465,4 | 54,9 | 183,3 | 21,6 | 124,2 | 14,6 | 75,6 | 8,9 | 848,4 | 100,0 |
|  | 2006 | 499,7 | 57,4 | 195,7 | 22,5 | 106,1 | 12,2 | 69,2 | 7,9 | 870,6 | 100,0 |
|  | 2007 | 486,7 | 57,8 | 182,5 | 21,7 | 105,5 | 12,5 | 67,3 | 8,0 | 842,2 | 100,0 |
|  | 2008 | 474,3 | 54,9 | 215,0 | 24,9 | 108,6 | 12,6 | 65,9 | 7,6 | 863,8 | 100,0 |
|  | 2009 | 469,5 | 58,3 | 160,7 | 19,9 | 90,9 | 11,3 | 84,6 | 10,5 | 805,8 | 100,0 |
|  | 2010 | 507,0 | 61,2 | 174,3 | 21,0 | 73,3 | 8,8 | 74,1 | 8,9 | 828,6 | 100,0 |
|  | 2011 | 543,2 | 64,3 | 170,6 | 20,2 | 69,0 | 8,2 | 62,0 | 7,3 | 844,7 | 100,0 |
|  | 2012 | 588,8 | 66,3 | 149,9 | 16,9 | 75,6 | 8,5 | 74,3 | 8,4 | 888,6 | 100,0 |
|  | 2013 | 621,4 | 70,3 | 118,4 | 13,4 | 73,0 | 8,3 | 70,9 | 8,0 | 883,8 | 100,0 |
|  | 2014 | 664,2 | 72,7 | 106,3 | 11,6 | 78,7 | 8,6 | 64,1 | 7,0 | 913,4 | 100,0 |
|  | 2015 | 693,6 | 73,7 | 103,3 | 11,0 | 68,0 | 7,2 | 76,1 | 8,1 | 941,0 | 100,0 |
|  | 2016 | 742,5 | 75,6 | 106,1 | 10,8 | 65,9 | 6,7 | 67,1 | 6,8 | 981,7 | 100,0 |
|  | 2017 | 875,0 | 76,3 | 129,7 | 11,3 | 65,8 | 5,7 | 76,3 | 6,7 | 1146,8 | 100,0 |
|  | 2018 | 913,1 | 77,9 | 110,5 | 9,4 | 71,7 | 6,1 | 76,1 | 6,5 | 1171,5 | 100,0 |
|  | 2019 | 1064,6 | 77,7 | 106,9 | 7,8 | 97,8 | 7,1 | 100,3 | 7,3 | 1369,7 | 100,0 |
|  | 2020 | 1010,4 | 77,7 | 127,8 | 9,8 | 74,6 | 5,7 | 87,1 | 6,7 | 1300,0 | 100,0 |
|  | 2021 | 1077,6 | 76,3 | 146,8 | 10,4 | 85,3 | 6,0 | 101,8 | 7,2 | 1411,5 | 100,0 |
|  | 2022 | 1019,6 | 73,6 | 160,8 | 11,6 | 87,9 | 6,3 | 117,6 | 8,5 | 1385,9 | 100,0 |
|  | **Total** | **14891,7** | **65,9** | **3538,6** | **15,7** | **2448,4** | **10,8** | **1712,9** | **7,6** | **22591,5** | **100,0** |
| **Female** | 2000 | 86,9 | 32,3 | 97,4 | 36,2 | 56,8 | 21,1 | 28,0 | 10,4 | 269,0 | 100,0 |
|  | 2001 | 114,2 | 34,7 | 143,8 | 43,7 | 47,9 | 14,6 | 23,4 | 7,1 | 329,3 | 100,0 |
|  | 2002 | 122,0 | 39,4 | 120,6 | 38,9 | 37,7 | 12,2 | 29,5 | 9,5 | 309,8 | 100,0 |
|  | 2003 | 118,8 | 40,0 | 118,3 | 39,8 | 40,3 | 13,5 | 19,8 | 6,7 | 297,2 | 100,0 |
|  | 2004 | 129,1 | 43,5 | 116,1 | 39,1 | 27,4 | 9,2 | 24,3 | 8,2 | 296,9 | 100,0 |
|  | 2005 | 116,0 | 38,2 | 128,5 | 42,4 | 23,6 | 7,8 | 35,3 | 11,6 | 303,4 | 100,0 |
|  | 2006 | 121,5 | 41,5 | 122,9 | 42,0 | 21,3 | 7,3 | 26,8 | 9,2 | 292,4 | 100,0 |
|  | 2007 | 104,5 | 38,3 | 119,7 | 43,9 | 26,5 | 9,7 | 21,8 | 8,0 | 272,6 | 100,0 |
|  | 2008 | 111,5 | 37,3 | 141,7 | 47,4 | 23,9 | 8,0 | 22,0 | 7,4 | 299,2 | 100,0 |
|  | 2009 | 104,2 | 45,1 | 86,4 | 37,4 | 12,4 | 5,4 | 28,0 | 12,1 | 231,0 | 100,0 |
|  | 2010 | 110,3 | 43,8 | 97,7 | 38,8 | 17,6 | 7,0 | 26,1 | 10,4 | 251,8 | 100,0 |
|  | 2011 | 140,8 | 49,8 | 101,1 | 35,8 | 10,4 | 3,7 | 30,1 | 10,7 | 282,5 | 100,0 |
|  | 2012 | 135,7 | 49,7 | 86,8 | 31,8 | 15,3 | 5,6 | 35,3 | 12,9 | 273,1 | 100,0 |
|  | 2013 | 158,3 | 60,5 | 60,5 | 23,1 | 11,8 | 4,5 | 31,2 | 11,9 | 261,7 | 100,0 |
|  | 2014 | 173,8 | 62,1 | 63,5 | 22,7 | 17,0 | 6,1 | 25,4 | 9,1 | 279,8 | 100,0 |
|  | 2015 | 197,2 | 68,3 | 53,4 | 18,5 | 13,8 | 4,8 | 24,4 | 8,5 | 288,8 | 100,0 |
|  | 2016 | 197,2 | 68,2 | 56,2 | 19,4 | 11,8 | 4,1 | 23,9 | 8,3 | 289,1 | 100,0 |
|  | 2017 | 261,7 | 68,8 | 72,4 | 19,0 | 18,9 | 5,0 | 27,2 | 7,2 | 380,2 | 100,0 |
|  | 2018 | 276,6 | 71,1 | 66,1 | 17,0 | 15,7 | 4,0 | 30,8 | 7,9 | 389,1 | 100,0 |
|  | 2019 | 290,7 | 71,5 | 62,5 | 15,4 | 20,6 | 5,1 | 32,9 | 8,1 | 406,6 | 100,0 |
|  | 2020 | 293,0 | 71,8 | 64,5 | 15,8 | 16,3 | 4,0 | 34,4 | 8,4 | 408,3 | 100,0 |
|  | 2021 | 357,0 | 70,5 | 91,0 | 18,0 | 18,0 | 3,6 | 40,6 | 8,0 | 506,7 | 100,0 |
|  | 2022 | 306,2 | 64,6 | 94,1 | 19,9 | 20,8 | 4,4 | 52,8 | 11,1 | 473,9 | 100,0 |
|  | **Total** | **4027,2** | **54,5** | **2165,1** | **29,3** | **526,0** | **7,1** | **674,0** | **9,1** | **7392,3** | **100,0** |

(Continues…)

(Continuation…)

| **Sex** | **Year** | | **Hanging** | | | | **Poisoning** | | | | **Firearm** | | | | **Other** | | | | **Total** | | | |
| --- | --- | --- | --- | --- | --- | --- | --- | --- | --- | --- | --- | --- | --- | --- | --- | --- | --- | --- | --- | --- | --- | --- |
|  |  |  | **N** | | **%** | | **N** | | **%** | | **N** | | **%** | | **N** | | **%** | | **N** | | **%** | |
| **Male** | 2000 | 243,6 | | 51,3 | | 56,2 | | 11,8 | | 142,3 | | 30,0 | | 32,7 | | 6,9 | | 474,7 | | 100,0 | |  |
|  | 2001 | 342,2 | | 55,8 | | 56,4 | | 9,2 | | 169,4 | | 27,6 | | 45,6 | | 7,4 | | 613,6 | | 100,0 | |  |
|  | 2002 | 314,8 | | 54,5 | | 79,7 | | 13,8 | | 147,3 | | 25,5 | | 35,7 | | 6,2 | | 577,5 | | 100,0 | |  |
|  | 2003 | 348,7 | | 61,0 | | 52,0 | | 9,1 | | 141,3 | | 24,7 | | 29,1 | | 5,1 | | 571,1 | | 100,0 | |  |
|  | 2004 | 354,9 | | 64,0 | | 49,4 | | 8,9 | | 115,6 | | 20,9 | | 34,4 | | 6,2 | | 554,3 | | 100,0 | |  |
|  | 2005 | 349,4 | | 64,1 | | 54,7 | | 10,0 | | 100,6 | | 18,5 | | 40,3 | | 7,4 | | 545,0 | | 100,0 | |  |
|  | 2006 | 378,2 | | 65,4 | | 72,8 | | 12,6 | | 84,8 | | 14,7 | | 42,4 | | 7,3 | | 578,2 | | 100,0 | |  |
|  | 2007 | 382,2 | | 67,1 | | 62,8 | | 11,0 | | 79,0 | | 13,9 | | 45,5 | | 8,0 | | 569,6 | | 100,0 | |  |
|  | 2008 | 362,7 | | 64,2 | | 73,3 | | 13,0 | | 84,7 | | 15,0 | | 43,9 | | 7,8 | | 564,6 | | 100,0 | |  |
|  | 2009 | 365,3 | | 63,6 | | 74,4 | | 12,9 | | 78,6 | | 13,7 | | 56,6 | | 9,8 | | 574,8 | | 100,0 | |  |
|  | 2010 | 396,7 | | 68,8 | | 76,6 | | 13,3 | | 55,7 | | 9,7 | | 47,9 | | 8,3 | | 576,9 | | 100,0 | |  |
|  | 2011 | 402,4 | | 71,6 | | 69,5 | | 12,4 | | 58,5 | | 10,4 | | 31,9 | | 5,7 | | 562,2 | | 100,0 | |  |
|  | 2012 | 453,1 | | 73,6 | | 63,1 | | 10,2 | | 60,4 | | 9,8 | | 39,0 | | 6,3 | | 615,5 | | 100,0 | |  |
|  | 2013 | 463,2 | | 74,5 | | 57,9 | | 9,3 | | 61,2 | | 9,8 | | 39,7 | | 6,4 | | 622,1 | | 100,0 | |  |
|  | 2014 | 490,4 | | 77,4 | | 42,8 | | 6,8 | | 61,7 | | 9,7 | | 38,7 | | 6,1 | | 633,6 | | 100,0 | |  |
|  | 2015 | 496,5 | | 76,1 | | 49,9 | | 7,7 | | 54,1 | | 8,3 | | 51,7 | | 7,9 | | 652,2 | | 100,0 | |  |
|  | 2016 | 545,3 | | 78,7 | | 49,9 | | 7,2 | | 54,1 | | 7,8 | | 43,2 | | 6,2 | | 692,6 | | 100,0 | |  |
|  | 2017 | 613,4 | | 80,0 | | 57,3 | | 7,5 | | 46,9 | | 6,1 | | 49,0 | | 6,4 | | 766,6 | | 100,0 | |  |
|  | 2018 | 636,5 | | 81,4 | | 44,4 | | 5,7 | | 56,1 | | 7,2 | | 45,3 | | 5,8 | | 782,3 | | 100,0 | |  |
|  | 2019 | 773,9 | | 80,4 | | 44,4 | | 4,6 | | 77,2 | | 8,0 | | 67,4 | | 7,0 | | 963,0 | | 100,0 | |  |
|  | 2020 | 717,4 | | 80,5 | | 63,3 | | 7,1 | | 58,3 | | 6,5 | | 52,7 | | 5,9 | | 891,7 | | 100,0 | |  |
|  | 2021 | 720,5 | | 79,6 | | 55,8 | | 6,2 | | 67,3 | | 7,4 | | 61,2 | | 6,8 | | 904,8 | | 100,0 | |  |
|  | 2022 | 713,4 | | 78,2 | | 66,7 | | 7,3 | | 67,1 | | 7,4 | | 64,9 | | 7,1 | | 912,0 | | 100,0 | |  |
|  |  | **10864,5** | | **71,5** | | **1373,5** | | **9,0** | | **1922,4** | | **12,6** | | **1038,9** | | **6,8** | | **15199,2** | | **100,0** | |  |
