# Supplementary figures and images for: Adolescent suicide trends in Brazil (2000–2022): An ecological analysis by sex, age, and suicide methods
Source: PLoS One. 2025 Jul 18;20(7):e0309505. doi: 10.1371/journal.pone.0309505 (PMC12273947; doi:10.1371/journal.pone.0309505)

Supplementary Figure 1. Redistribution of deaths classified as Garbage Codes.

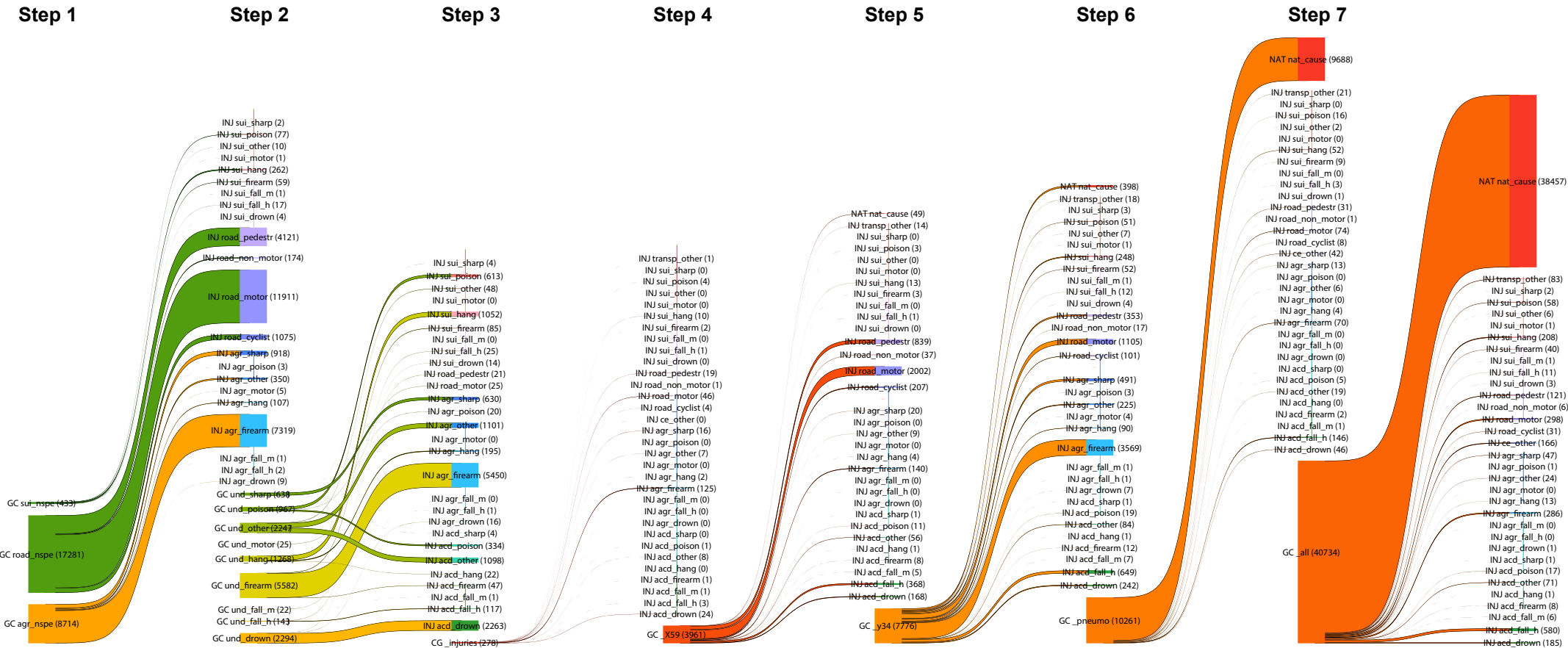

Supplement: S1 Fig — (PDF) [file pone.0309505.s002.pdf]
